# Effects of new *Torulaspora delbrueckii* killer yeasts on the must fermentation kinetics and aroma compounds of white table wine

**DOI:** 10.3389/fmicb.2015.01222

**Published:** 2015-11-03

**Authors:** Rocío Velázquez, Emiliano Zamora, María L. Álvarez, Luis M. Hernández, Manuel Ramírez

**Affiliations:** ^1^Departamento de Ciencias Biomédicas (Área de Microbiología), Facultad de Ciencias, Universidad de ExtremaduraBadajoz, Spain; ^2^Estación Enológica, Junta de ExtremaduraAlmendralejo, Spain

**Keywords:** *Torulaspora delbrueckii*, yeast, killer, must fermentation, winemaking, white table wine, aroma compounds

## Abstract

*Torulaspora delbrueckii* is becoming widely recommended for improving some specific characteristics of wines. However, its impact on wine quality is still far from satisfactory at the winery level, mostly because it is easily replaced by *Saccharomyces cerevisiae*-like yeasts during must fermentation. New *T. delbrueckii* killer strains were here isolated and selected for winemaking. They killed *S. cerevisiae* yeasts and were able to dominate and complete the fermentation of sterile grape must. Sequential yeast inoculation of non-sterile white must with *T. delbrueckii* followed by *S. cerevisiae* did not ensure *T. delbrueckii* dominance or wine quality improvement. Only a single initial must inoculation at high cell concentrations allowed the *T. delbrueckii* killer strains to dominate and complete the must fermentation to reach above 11% ethanol, but not the non-killer strains. None of the wines underwent malolactic fermentation as long as the must had low turbidity and pH. Although no statistically significant differences were found in the wine quality score, the *S. cerevisiae*-dominated wines were preferred over the *T. delbrueckii*-dominated ones because the former had high-intensity fresh fruit aromas while the latter had lower intensity, but nevertheless nice and unusual dried fruit/pastry aromas. Except for ethyl propanoate and 3-ethoxy-1-propanol, which were more abundant in the *T. delbrueckii*–dominated wines, most of the compounds with fresh fruit odor descriptors, including those with the greatest odor activity values (isoamyl acetate, ethyl hexanoate, and ethyl octanoate), were more abundant in the *S. cerevisiae*–dominated wines. The low relative concentrations of these fruity compounds made it possible to detect in the *T. delbrueckii*–dominated wines the low-relative-concentration compounds with dried fruit and pastry odors. An example was γ-ethoxy-butyrolactone which was significantly more abundant in these wines than in those dominated by *S. cerevisiae*.

## Introduction

The non-*Saccharomyces* yeasts which are usually present in spontaneous must fermentations have been receiving ever more attention by the part of wine microbiologists because some of them can improve wine complexity. The yeasts which have lately been investigated for wine quality improvement belong to *Candida, Kloeckera, Hanseniaspora, Zygosaccharomyces, Schizosaccharomyces, Torulaspora, Brettanomyces, Saccharomycodes, Pichia*, and *Williopsis* genera (Jolly et al., 2006). Among them, *Torulaspora delbrueckii* is probably the most commonly used in winemaking. Controlled inoculation with this yeast is widely recommended for improving the complexity and for enhancing certain specific characteristics of wines (Jolly et al., 2006; Bely et al., 2008; Renault et al., 2009; Azzolini et al., 2012, 2015). This yeast can also be used to increase glycerol (Contreras et al., 2015) and mannoproteins (Comitini et al., 2011; Belda et al., 2015), or to reduce ethanol (Contreras et al., 2015) in the wine. However, its commercial impact on wine quality is still far from satisfactory, mostly because of the difficulty in reliably controlling the desired participating proportion of *T. delbrueckii* with respect to the other wine yeast species involved in the same must fermentation process, mainly *Saccharomyces cerevisiae*-like yeasts. It has been reported that the mixed inoculation of *T. delbrueckii* and *S. cerevisiae* reduces such off-flavor compounds as volatile acidity, acetaldehyde, and acetoin (Herraiz et al., 1990; Ciani et al., 2006; Bely et al., 2008), and leads to a systematic increase of 2-phenylethanol, terpenols, and lactones (Herraiz et al., 1990; Comitini et al., 2011; Azzolini et al., 2012; Sadoudi et al., 2012). However, results concerning ester production remain confusing. It has been reported that mixed inoculation can increase the total ester concentration (in particular that of isoamyl acetate and ethyl hexanoate, octanoate, and 3-hydroxybutanoate) relative to pure-culture inoculation (Herraiz et al., 1990). But the contrary has also been reported, i.e., that the total ester concentration of mixed inoculations was less than that of a pure *S. cerevisiae* culture, with a significant reduction in acetate esters, in particular of isoamyl acetate (Comitini et al., 2011; Sadoudi et al., 2012). Similarly, no difference in the overall ester concentrations was found between mixed *T. delbrueckii*/*S. cerevisiae* and single *S. cerevisiae* inoculation, although the level of some esters (ethyl 3-hydroxybutanoate, for instance) was higher in the mixed culture while that of others (such as isoamyl acetate) was lower (Azzolini et al., 2012). These apparently contradictory results concerning ester concentrations may depend on the proportion of each yeast species during must fermentation, or also on the eventual occurrence of malolactic fermentation, neither of which possibilities were discussed in any depth by those authors. Additionally, it has been shown that ester production by *T. delbrueckii* is strain dependent, and that the aromas resulting from this yeast can differ when it is associated with *S. cerevisiae* in mixed cultures (Renault et al., 2009).

As most non-*Saccharomyces* yeasts, *T. delbrueckii* has less fermentation vigor and a slower growth rate than *S. cerevisiae* under usual wine fermentation conditions, being quickly overcome by wild or inoculated *S. cerevisiae* strains (Mauricio et al., 1998; González-Royo et al., 2014). Thus, knowledge about the interactions between *Saccharomyces* and *Torulaspora* wine yeasts during wine fermentation needs to be improved to better predict the relative participation of each yeast species (Ciani et al., 2010). The availability of good-fermenting killer *T. delbrueckii* strains, able to kill the omnipresent wild *Saccharomyces* yeasts or to control the excessive growth of inoculated *S. cerevisiae* strains, could be an interesting tool with which to attain the desired domination of each inoculated yeast during must fermentation, and thus result in improved quality of the wine. The isolation of *T. delbrueckii* killer strains has been described previously (Sangorrin et al., 2007), but they have not been used and analyzed in depth for winemaking as it has been *S. cerevisiae* K2 strains (Pérez et al., 2001). The effect of *S. cerevisiae* killer strains on the growth of sensitive strains during must fermentation was seen to depend on the initial proportion of killer yeasts, the susceptibility of sensitive strains, and the treatment of the must. An initial proportion of 2–6% killer yeasts was enough to suppress isogenic sensitive strains in sterile filtered must, although a greater initial proportion of killer yeasts may be needed to get the same effect against non-isogenic strains. The suspended solids that remain in the must after cold-settling were seen to reduce the killer toxin effect due to inactivation by absorption onto the grape particles (Pérez et al., 2001).

The objective of the present work was to evaluate the use of new killer *T. delbrueckii* strains (Kbarr) for white wine making. We addressed the following issues: (i) capacity of Kbarr strains to dominate and complete must fermentation in the presence of *S. cerevisiae* yeasts; (ii) influence of must treatment on this Kbarr-1 strain domination; (iii) influence of Kbarr strains on malolactic fermentation; and (iv) analysis of the aroma profile of *T. delbrueckii* white wine as compared with *S. cerevisiae* white wine. The usefulness of killer *T. delbrueckii* strains for winemaking will be discussed.

## Materials and Methods

### Yeast Strains and Culture Media

EX85, EX85R, and E7AR1 are prototrophic and homothallic *S. cerevisiae* wine yeasts previously isolated from Spanish wineries, selected for winemaking (Regodón et al., 1997; Ramírez et al., 1998), and sold by Heral Enología SL (Almendralejo, Spain). EX85 is K2-killer, EX85R is virus-free killer-sensitive cycloheximide-resistant (cyh^R^), and E7AR1 is K2-killer cyh^R^. The *S. cerevisiae* K2-killer strains kill other killer-sensitive *S. cerevisiae* strains but do not kill *T. delbrueckii* yeasts. The new *T. delbrueckii* Kbarr wine yeasts are prototrophic strains isolated from spontaneous fermentations of grapes from vineyards of the Albarregas (*Barraecas* in Latin) river valley in Spain. They kill all *S. cerevisiae* killer and non-killer strains and the non-killer *T. delbrueckii* strains. The industrial use of these Kbarr yeasts is under patent application. The yeast strains used in this work are summarized in **Table 1**.

**Table 1:** Yeast strains used.

Strain	Genotype/Relevant phenotype	Origin
*Sc* EX85	*MAT a/α HO/HO* L-A M-2 [K2^+^]	M. Ramírez^a^ (from wine)
*Sc* EX85R	*MAT a/α HO/HO CYH2^R^/cyh2^S^* [cyh^R^ K2^0^]	M. Ramírez^a^
*Sc* E7AR1	*MAT a/α HO/HO CYH2^R^/cyh2^S^* [K2^+^]	M. Ramírez^a^
*Td* EX1180	*wt* L-A M-barr-1 [Kbarr-1^+^]	This study (from wine)
*Td* EX1180-11C4	*cyh^R^* L-Abarr M-barr-1 [cyh^R^ Kbarr-1^+^]	This study (from EX1180)
*Td* EX1180-2K^-^	*cyh^R^* L-Abarr M-barr-0 [cyh^R^ Kbarr^0^]	This study (from EX1180)
*Td* EX1257	*wt* L-Abarr M-barr-2 [Kbarr-2^+^]	This study (from wine)
*Td* EX1257-CYH5	*cyh^R^* L-Abarr M-barr-2 [cyh^R^ Kbarr-2^+^]	This study (from EX1257)

*^a^M. Ramírez, Departamento de Ciencias Biomédicas, Universidad de Extremadura, Badajoz, Spain. Sc, Saccharomyces cerevisiae; Td, Torulaspora delbrueckii.*

YEPD + cycloheximide (cyh) is YEPD-agar (1% Bacto-yeast extract, 2% Bacto-peptone, 2% glucose, 2% Bacto-agar) supplemented with cyh, prepared in a concentrated ethanol solution to a final concentration of 2 μg/mL (Pérez et al., 2000). Standard yeast genetics procedures were used for sporulation (Kaiser et al., 1994). Cells were grown on YEPD plates for 2 days at 30°C, transferred to sporulation plates (1% potassium acetate, 0.1% Bacto-yeast extract, 0.05% glucose, 2% Bacto-agar) and incubated for 7–30 days at 25°C.

### Determination of Yeast Killer Activity

Killer activity was tested on low-pH (pH 3.3 or 4.0) methylene blue plates (3.3MB or 4.0MB; Kaiser et al., 1994) seeded with 100 μL of a 48-h grown culture of the sensitive strain (Ramírez et al., 2004). Depending on the experiments, the strains being tested for killer activity were either loaded as 4 μL aliquots of stationary phase cultures, patched from solid cultures, or replica plated onto the seeded MB plates. Then the plates were incubated for 4–8 days at 12 or 20°C.

### Laboratory Must Fermentation

Must fermentation was carried out in 5-L Erlenmeyer flasks with 3.5 L of Cigüente grape must (18.0°Brix, pH 3.5, 50 mg/L SO_2_, and 0.3 g/L Actimax nutrients from Productos Agrovin S.A.) sterilized by membrane filtration through a Millipore system (0.45-μm membrane). Yeast cells were cultured in YEPD broth for 2 days at 30°C, washed twice (by centrifugation) with sterile water, and suspended in the must at the desired concentration. Fermentations were conducted at 18°C for 20 days. Yeast growth (determination of total yeast cells by counting with a Neubauer chamber, and viable cells by counting the yeast colonies that arose on YEPD-agar plates), and the °Brix were monitored. All experiments were done in triplicate.

### Winery Vinification Trials

The yeast inocula were obtained in a pilot plant of the company Heral Enología SL following its industrial procedure. Cells were cultured in beet molasses broth [5% beet molasses, 0.2% Bacto-yeast extract, 0.075% (NH_4_)_2_HPO_4_, 0.1% MgSO_4_⋅7H_2_O, adjusted to pH 3.5 with HCl] for 18 h at 30°C with strong aeration, washed twice (by centrifugation) with sterile distilled water, and inoculated in 350-L stainless steel tanks with cold-settled white Cigüente (19.0–19.8°Brix, pH 3.42, 80–250 NTU, 50 mg/L SO_2_, and 0.3 g/L Actimax) or Macabeo (20.4–20.8°Brix, pH 3.29–3.55, 80–250 NTU, 50 mg/L SO_2_, and 0.3 g/L Actimax) grape must to a final concentration of 2–4 × 10^6^ cells/mL for *S. cerevisiae* and 2–4 × 10^7^ cells/mL for *T. delbrueckii*. The vinification process was conducted at 16–18°C. The density, °Brix, and yeast growth (total and viable yeast cells) were monitored throughout fermentation. The tanks were hermetically closed when reducing sugars reached around 1% to avoid oxidation problems. At the end of fermentation, the settled solids were discarded. An 800-mL centrifuged sample of each wine was taken for the analytical assays. The wines were stored at 12°C. After 30 days following the beginning of fermentation, settled solids were again discarded, a 2-L sample of each wine was taken for the first aroma compounds and organoleptic assays, and the wines were returned to store at 12°C. At 60 days, settled solids were discarded once again and the second aroma compounds and organoleptic assays were carried out. The organoleptic characteristics (flavor, color, and odor) of the wines were tested by a panel of 12 experts. Wines were presented in clear tulip-shaped wine glasses covered with glass Petri dishes. A sample of 50–70 mL of wine was poured into each glass immediately before being analyzed by each judge. The temperature of the samples was from 10 to 13°C. Sensory profiles of wines were evaluated for overall aromatic complexity, and fresh fruit and dried fruit/pastry aroma intensities. The judges scored the quality of the wines on a six-point scale (0 = very poor, 1 = deficient, 2 = acceptable, 3 = good, 4 = very good, and 5 = excellent). The maximum score possible (60 points) was considered 100% preference. All experiments were done in duplicate.

### Determination of the Amount of Inoculated Yeasts during Must Fermentation

Determination of the percentage of genetically marked yeasts was done by the replica-plating method (Pérez et al., 2000). Samples from fermenting musts were diluted and plated onto YEPD-agar to obtain 100–300 colonies per plate. The detection of the cyh^R^ mutants was accomplished by replica-plating these plates to either YEPD + cyh (2 μg/mL) plates using sterile velvets and then to other plates of YEPD-agar to detect wild yeasts sensitive to cyh. The time needed to easily observe growth of resistant yeasts on YEPD + cyh at 30°C varied between 1 and 3 days depending on the yeast strain.

The percentage of wild parent yeasts, or genetically marked yeasts for the replica-plating results validation, was mostly determined by analyzing the mtDNA restriction pattern as previously described (Maqueda et al., 2010).

The yeast spore (after yeast growth on sporulation medium for 7–30 days at 25°C) or vegetative cell morphology were also eventually analyzed for validation of the previous results obtained by the replica-plating or mtDNA restriction pattern analyses. This morphology analysis was done by microscopic observation in a Nikon Eclipse 600 microscope equipped with a Nomarski 60× objective.

### Analytical Methods

Density, °Brix, pH, total acidity, volatile acid, reducing sugars, alcohol, and malic acid were determined according to the EC recommended methods (E.C, 1999). Lactic acid was determined using the EEC recommended method (E.E.C, 1990). Glycerol was determined with an enzymatic test (Roche, Germany). Mannoprotein content was measured as previously described (Quiros et al., 2012). T15 is the time needed to ferment 15% of the total sugars present in the must, and T100 is the time needed to ferment 100% of the total sugars (Ramírez et al., 1999).

The wine aroma compounds were analyzed by gas chromatography coupled to a mass detector. The minor aroma compounds were isolated and pre-concentrated following a solid-phase extraction (SPE) procedure (García-Carpintero et al., 2011). The analyses were carried out with an Agilent 6890 N gas chromatograph coupled to a Model 5973 mass detector and equipped with an autosampler. The column was a DB WAXETR (60 m × 0.25 mm, i.d; 0.25 μm film thickness). Quantitative data were obtained by calculating the peak area of each compound relative to that of the internal standard, interpolating with the corresponding calibration plot which had been constructed from the analysis of known amounts of the volatile aroma standards. For those compounds for which the authenticated standards were unavailable (ethyl 9-decenoate, diethyl 2-hydroxyglutarate, ethyl 2-hydroxy-3-phenylpropanoate, and γ-ethoxy-butyrolactone), the identification was based on spectral comparison with the Wiley A library data, and quantification was done using the calibration curves of standards with similar chemical structures obtained in the TIC mode. A total of 75 compounds were detected in the wines elaborated (Supplementary Table S1). The odor descriptor and the odor threshold concentration for each volatile compound were taken from the literature (Etievant and Maarse, 1991; Guth, 1997; Ferreira et al., 2000; Moyano et al., 2002; Zea et al., 2007; Muñoz et al., 2008; Pino and Queris, 2011). The odor activity value (OAV) is the ratio between the concentration of each individual aromatic compound and its odor threshold concentration (the minimal concentration that can be detected by the human nose). As no odor threshold concentration was available for some compounds, 1 mg/L was taken as the value for the ethyl 9-decenoate, ethyl 4-hydroxybutyrate, and 9-decenoic acid OAV calculations, and the value for γ-butyrolactone (0.035 mg/L) was taken for the γ-ethoxy-butyrolactone OAV calculation.

### Statistical Analysis

Data were analyzed for statistical significance by a one-way analysis of variance (ANOVA, *p* < 0.05) with the software package SPSS version 20.0 for Windows (Chicago, IL, USA).

## Results

### Effect of *T. delbrueckii* Killer Yeasts on the *S. cerevisiae* Population during Sterile-must Fermentation

The influence of any given yeast on winemaking will mostly depend on its ability to dominate the must fermentation while reducing the influence of the other participating yeasts. Complementary and reliable methods to monitor the different wine yeasts in the fermenting must are required to accurately determine the degree of domination of each yeast strain. We isolated and characterized new spontaneous cyh-resistant (cyh^R^) mutants from new *T. delbrueckii* killer yeasts that had previously been isolated and selected for winemaking (Regodón et al., 1997; Ramírez et al., 2015). Some of these mutants, such as EX1180-2K^-^ for instance, had lost the killer virus to become killer sensitive yeasts, but others, such as EX1180-11C4, retained the virus and the Kbarr-1 phenotype (**Table 1**). Both of these cyh^R^ mutant types had good must fermentation capabilities, and were easily monitored by simple replica-plating on YEPD-CYH agar. Additionally, these *T. delbrueckii* killer yeasts can also be distinguished from the always present *Saccharomyces* by analyzing their cell morphology, spore forming process, or mtDNA RFLPs (Supplementary Figure S1). Moreover, the killer phenotype or viral dsRNA analysis can also be used for this same purpose (not shown). These alternative techniques were satisfactorily used to validate the results obtained from the simple replica-plating assay on YEPD-CYH agar plates.

**FIGURE 1:**
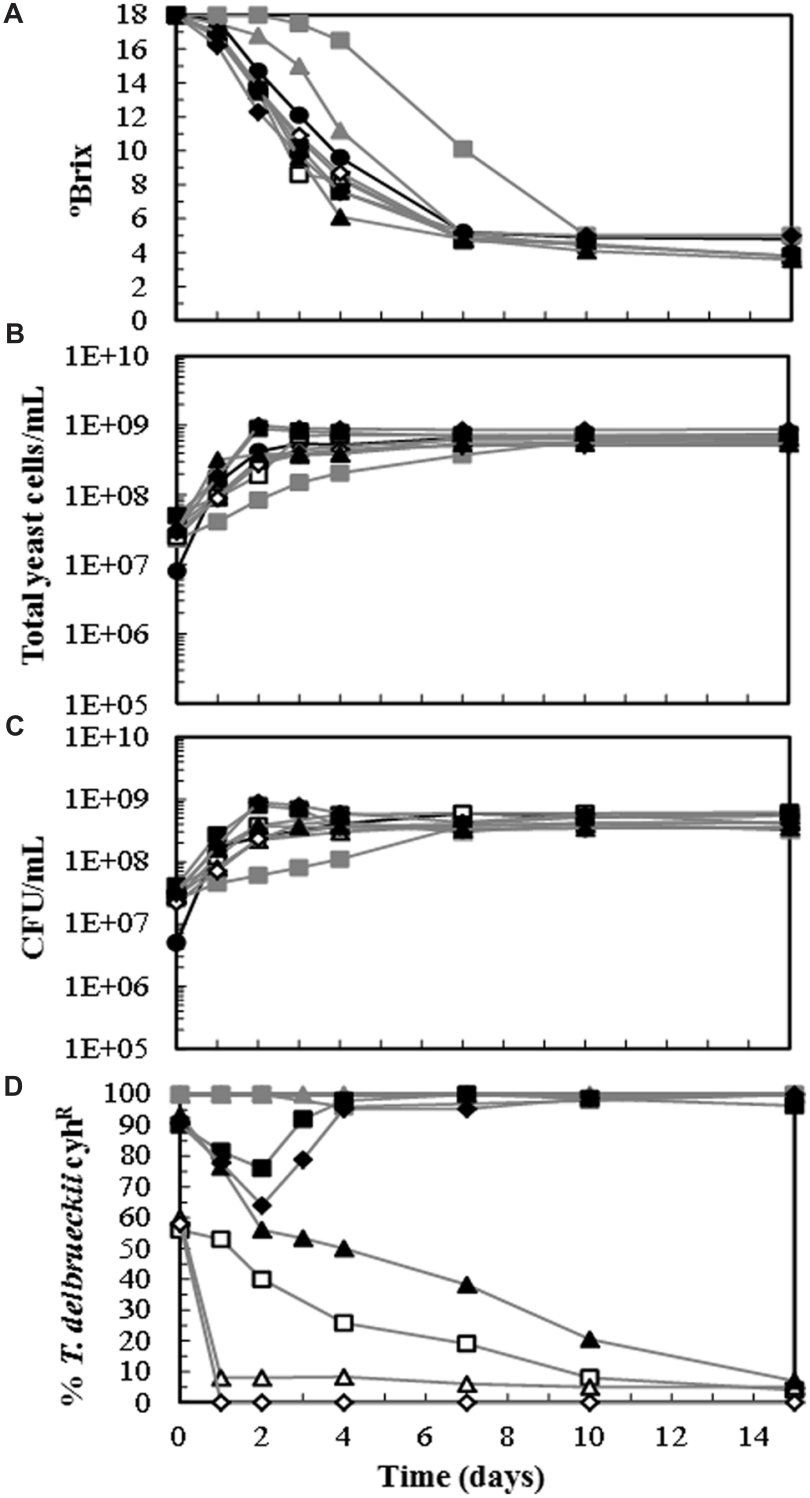
**Fermentation kinetic and yeast population dynamics of sterile-filtered and cold-settled Cigüente grape must inoculated with different yeast strains of *Saccharomyces cerevisiae*: EX85 (K2, cyh^S^), or *Torulaspora delbrueckii*: EX1180-11C4 (Kbarr-1, cyh^R^) and EX1180-2K^-^ (non-killer, cyh^R^). (A)** °Brix. **(B)** Total yeast cells. **(C)** Viable yeasts. **(D)** Percentage of *T. delbrueckii* cyh^R^ yeasts in each fermentation. Symbols: EX1180-11C4 in filtered musts (-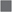-), EX1180-2K^-^ in filtered musts (-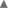-), EX85 (50%) + EX1180-11C4 (50%) in filtered musts (-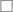-), EX85 (50%) + EX1180-11C4 (50%) cold-settled must (-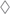-), EX85 (50%) + EX1180-2K^-^ (50%) in filtered musts (-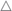-), EX85 (10%) + EX1180-11C4 (90%) in filtered musts (-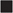-), EX85 (10%) + EX1180-11C4 (90%) cold-settled must (-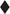-), and EX85 (10%) + EX1180-2K^-^ (90%) in filtered musts (-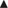-).

To determine whether the *T. delbrueckii* killer yeasts can dominate the must fermentation in the presence of *S. cerevisiae* wine strains, sterile-must laboratory micro vinifications were inoculated with both yeasts. Each yeast species was monitored through the process by replica-plating on YEPD-CYH and by the aforementioned complementary methods (mostly mtDNA RFLP analysis) in at least two samples for each vinification. The results of the different methods showed full agreement, supporting the utility of cyh^R^ as genetic marker to monitor *T. delbrueckii* in winemaking, as it was previously found for *S. cerevisiae* (Pérez et al., 2000; Ambrona et al., 2005, 2006). The must fermentation inoculated with *S. cerevisiae* alone or with two-yeast mixtures of *S. cerevisiae* + *T. delbrueckii* strains (one of them containing the cyh^R^ genetic marker) showed faster kinetics than those single-inoculated with a *T. delbrueckii* strain, although all fermentations were completed after 10 days (**Figure 1A**). The total yeast cell concentration increased to above 2 × 10^8^ cells/mL after 3–4 days from the start of fermentation, except for *T. delbrueckii* killer EX1180-11C4 which reached 2 × 10^8^ cells/mL after 7 days (**Figure 1B**). The number of viable cells increased in parallel with the number of total cells, except for the vinification of filtered must inoculated with EX85 (10%) + EX1180-11C4 (90%), and for that of cold-settled must inoculated with EX85 (10%) + EX1180-11C4 (90%). In both cases, a decrease in viable cells was observed between days 2 and 4 of fermentation (**Figure 1C**), indicating that the *S. cerevisiae* yeasts were killed by the *T. delbrueckii* killer yeasts. The *S. cerevisiae* EX85 strain dominated the must fermentation when initially combined with 50% of the non-killer *T. delbrueckii* EX1180-2K^-^ strain, that fell to 7% after 1 day of fermentation in filtered must (no grape particles present). This time required for *S. cerevisiae* EX85 domination was extended in filtered must fermentation when it was combined with the same initial proportion (50%) of the *T. delbrueckii* killer EX1180-11C4, which remained above 20% after 7 days. But this time was reduced again in cold-settled non-filtered must, where EX1180-11C4 disappeared after just 1 day (**Figure 1D**). A plausible explanation for this behavior is the presence of grape particles through the fermentation, which might adsorb and inactivate the toxin produced by *T. delbrueckii*, as it was previously shown for toxins produced by *S. cerevisiae* (Pérez et al., 2001). The *S. cerevisiae* EX85 strain also dominated the must fermentation when initially combined with 90% of the non-killer *T. delbrueckii* EX1180-2K^-^, although this latter strain remained at above 40% for 7 days in filtered must fermentation. On the contrary, the *S. cerevisiae* EX85 strain almost disappeared when initially combined with 90% of the killer *T. delbrueckii* EX1180-11C4, which was the dominating yeast throughout fermentation in filtered and in non-filtered grape must (**Figure 1D**).

### Winemaking with *T. delbrueckii* Killer and *S. cerevisiae* Yeasts

Once we had determined the *T. delbrueckii* cell concentration required to get its domination during must fermentation, new vinification trials were done using the commonest commercial recommendations: sequential yeast inoculation involving *T. delbrueckii* at the beginning (2–4 × 10^7^ CFU/mL) followed by *S. cerevisiae* (2–4 × 10^6^ CFU/mL) after 2 days of fermentation. In most vinifications, the *T. delbrueckii* viable population decreased to less than 10% of total viable yeast cells after around 1 day following *S. cerevisiae* inoculation. The wine obtained with these sequential mixed-yeast inoculations showed no relevant aromatic differences from those single-inoculated with a *S. cerevisiae* strain. This is probably because *S. cerevisiae*, which became the dominating yeast for most fermentation time, abolished the effect of *T. delbrueckii* on the wine aromatic compounds during the first two fermentation days. Sometimes, the *S. cerevisiae* domination was slower and less efficient, remaining more than 30% of *T. delbrueckii* killer yeasts at the end of a very slow fermentation. These fermentations were frequently not fully completed, mainly in those wines with ethanol concentrations greater than 11.5%. Therefore, in these cases, the wines obtained were not dry since they contained more than 6 g/L of reducing sugars.

**FIGURE 2:**
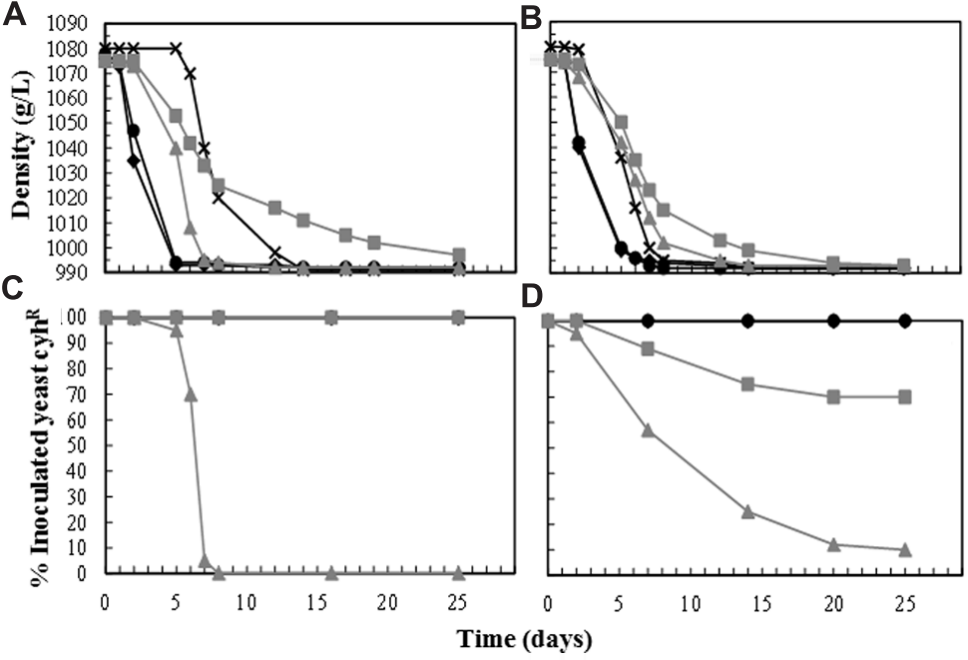
**Must fermentation kinetics and yeast population dynamics of two independent sets of vinification trials done with two Macabeo grape musts (<100 NTU, pH < 3.5) in 2011 **(A,C)** and 2012 **(B,D)**.** Each yeast was single inoculated in the fresh must at a cell concentration of 2–4 × 10^6^ CFU/mL for the *S. cerevisiae* strains E7AR1 (K2, cyh^R^) or EX85R (non-killer, cyh^R^), and 2–4 × 10^7^ CFU/mL for *T. delbrueckii* strains EX1180-11C4 (Kbarr-1, cyh^R^) or EX1180-2K^-^ (non-killer, cyh^R^). **(A,B)** Evolution of must-wine density. **(C,D)** Evolution of the percentage of each inoculated yeast (cyh^R^) during the must fermentation. Symbols: Non-inoculated control (-**×**-), E7AR1 (-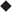-), EX85R (-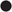-), EX1180-11C4 (-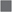-), and EX1180-2K^-^ (-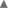-).

In view of these disappointing results, new vinification trials were performed using single inoculation with *T. delbrueckii* (2–4 × 10^7^ CFU/mL). The *S. cerevisiae* yeasts present in these fermentations were only those coming into the fresh cold-settled white must (less than 10^5^ CFU/mL). This must was very well clarified (less than 100 NTU turbidity) and its pH was corrected to 3.3 by the addition of tartaric acid. As controls for comparison, vinifications were also performed using only a single initial inoculation with *S. cerevisiae* (2–4 × 10^6^ CFU/mL). All the grape musts contained around 11°Be, but less than 11.5°Be to avoid the toxic effect of ethanol on *T. delbrueckii* yeasts and to facilitate the completion of fermentation. The fastest fermentations were always those inoculated with *S. cerevisiae*, while those inoculated with *T. delbrueckii* started quickly but slowed down as the ethanol concentration increased, and were very slow by the end of fermentation. Non-inoculated fermentations, performed mostly by wild *Saccharomyces* yeasts from the must, were the slowest in starting, but they finished the fermentation before the vinifications single-inoculated with *T. delbrueckii* yeasts (**Figures 2A,B**; **Table 2**). The inoculated *S. cerevisiae* dominated the fermentations (100%) from the beginning to the end. The *T. delbrueckii* killer strains also dominated the fermentation, although sometimes their proportion decreased to 75% at the end of fermentation (wine density less than 995 g/L, **Figures 2C,D**). These wines contained a certain amount of reducing sugars (5.98 ± 2.15), especially when this *T. delbrueckii*-domination was 100% throughout fermentation and no *S. cerevisiae* ethanol-resistant wild yeasts were present at the end of the process. *T. delbrueckii* non-killer strain did not dominate the fermentation. Sometimes its proportion decreased quickly to less than 1% or, after decreasing, it remained at a proportion of around 10%. All these *T. delbrueckii*-non-dominated fermentations were completed, and they were faster than those dominated by *T. delbrueckii* because *S. cerevisiae* ethanol-resistant wild yeasts were always present in increasing proportions (**Figure 2**). The main fermentation aroma of these *T. delbrueckii*-non-dominated vinifications was fresh fruit, while it was cooked/dried fruit and pastry for the vinifications dominated by *T. delbrueckii* killer yeasts. The main aroma of the non-inoculated control and the non-killer *T. delbrueckii* inoculated vinifications was very similar to those single-inoculated and dominated by *S. cerevisiae*, although the latter had greater fresh-fruit odor intensities.

**Table 2:** Must fermentation parameters and white wine analysis results of independent winery vinifications made with Cigüente and Macabeo musts and of an ANOVA to study the effect of single initial inoculation with *S. cerevisiae* or *T. delbrueckii* yeasts.

Parameter	Yeast species		*p*^a^
	*S. cerevisiae*	*T. delbrueckii*	
T15 (days)	1.75 ± 0.23	5.13 ± 0.60	0.000
T100 (days)	10.0 ± 3.87	20.7 ± 3.08	0.043
Preference (%)	64.4 ± 4.67	56.7 ± 3.79	0.128
Frequency in TF (%)	100 ± 0.00	96.1 ± 2.23	0.180
Frequency in EF (%)	100 ± 0.00	86.8 ± 7.49	0.172
Alcohol (% v/v)	11.3 ± 0.58	11.2 ± 0.56	0.967
Glycerol (g/L)	6.1 ± 0.20	5.65 ± 0.37	0.315
pH	3.07 ± 0.07	3.20 ± 0.05	0.165
Total acidity (g/L)	7.21 ± 0.23	6.89 ± 0.23	0.362
Volatile acidity (g/L)	0.26 ± 0.05	0.36 ± 0.07	0.366
Density (g/L)	990.7 ± 0.41	994.6 ± 1.25	0.026
Reducing sugars (g/L)	1.24 ± 0.15	5.98 ± 2.15	0.091
Mannoproteins (mg/L)	58.8 ± 4.74	123.3 ± 32.6	0.086
Malic acid (g/L)	1.47 ± 0.12	1.45 ± 0.16	0.926
Lactic acid (g/L)	0.07 ± 0.01	0.14 ± 0.06	0.363

*The data are the mean ± standard error of 11 independent experiments done with *S. cerevisiae* and 12 with *T. delbrueckii*. ^a^*p*-values obtained by ANOVA for the wines made with each yeast species. TF, tumultuous fermentation; EF, end of fermentation; T15, time needed to ferment 15% of the total sugars present in the must; T100, time needed to ferment 100% of the total sugars or to get a nonfluctuating level under 8 g/L*.

**FIGURE 3:**
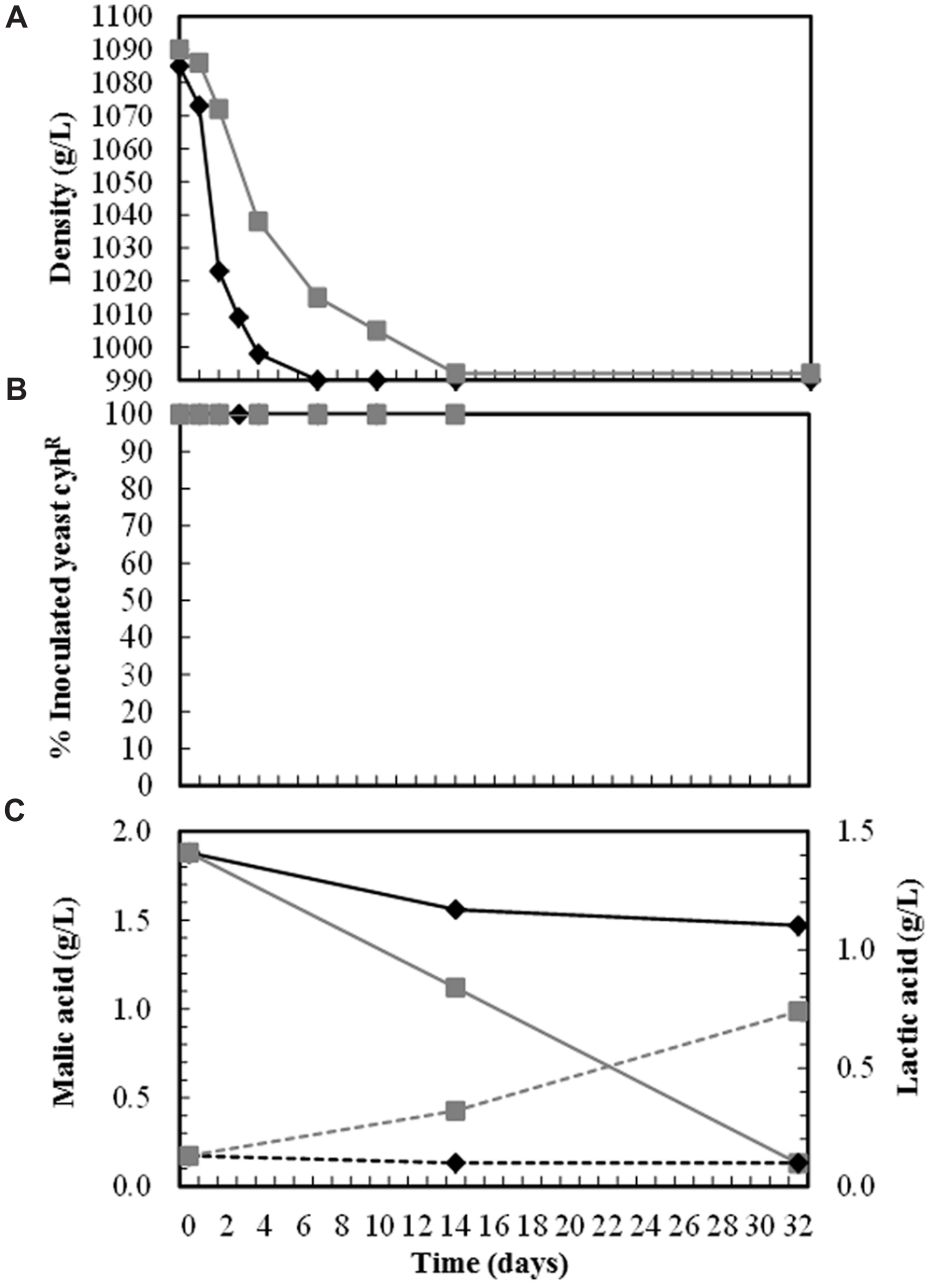
**Must fermentation kinetics **(A)**, yeast population dynamics **(B)**, and malic/lactic acid degradation/production during the vinification trials done with turbid cold-settled Macabeo grape musts (around 250 NTU, pH 3.55).** Each yeast was single inoculated in the fresh must at a cell concentration of 2–4 × 10^6^ CFU/mL for the *S. cerevisiae* strain E7AR1 (K2, cyh^R^), and 2–4 × 10^7^ CFU/mL for *T. delbrueckii* strain EX1180-11C4 (Kbarr-1, cyh^R^). Symbols: E7AR1 (-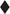-), EX1180-11C4 (-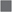-), malic acid (—), and lactic acid (- - - -).

None of these wines underwent malolactic fermentation, even those inoculated with *T. delbrueckii* killer strains that had slow fermentation kinetics and more than 5 g/L of reducing sugars (**Table 2**), conditions that usually favor the growth of lactic acid bacteria. However, the wines elaborated with the same grape must but of greater turbidity (around 250 NTU) and higher pH (3.55) did undergo malolactic fermentation (malic acid decreased, while lactic acid increased) when single-inoculated with *T. delbrueckii* killer yeasts, but not when single-inoculated with *S. cerevisiae* yeasts. Although both inoculated yeasts dominated the fermentation (100%) throughout the process, the fermentation inoculated with *S. cerevisiae* was faster than that inoculated with *T. delbrueckii* killer yeast (taking 7 and 14 days to complete fermentation, respectively; **Figure 3**).

### Organoleptic and Physicochemical Analysis of the Wines

The wines made with *S. cerevisiae* or *T. delbrueckii* for which these yeasts dominated all or most of the fermentation process were compared. In particular, the wines inoculated with non-killer *T. delbrueckii* yeast that became replaced by wild *S. cerevisiae* yeast and those that underwent malolactic fermentation were not considered for this analysis. The wine parameter values were consistent with both wine types being non-defective, good-quality products. Significant differences were only found for the fermentation kinetics parameters (T15 and T100), wine density, and marginally significant differences for the amount of reducing sugars and mannoproteins (**Table 2**). However, although no statistically significant differences were found in the organoleptic quality score, the *S. cerevisiae*-dominated wines were preferred over the *T. delbrueckii*-dominated wines because the former had high-intensity fresh fruity aromas. The *T. delbrueckii*-dominated wines had low-intensity fresh fruit aroma, better flavor complexity, nice but unusual dried fruit (cooked fruit, pastry, and candy) aromas, a little sourness, and some aged/evolved taste. These unusual wine aromas were very similar to the aromas detected during the respective must fermentations of the same wines (see above), but less intense.

**FIGURE 4:**
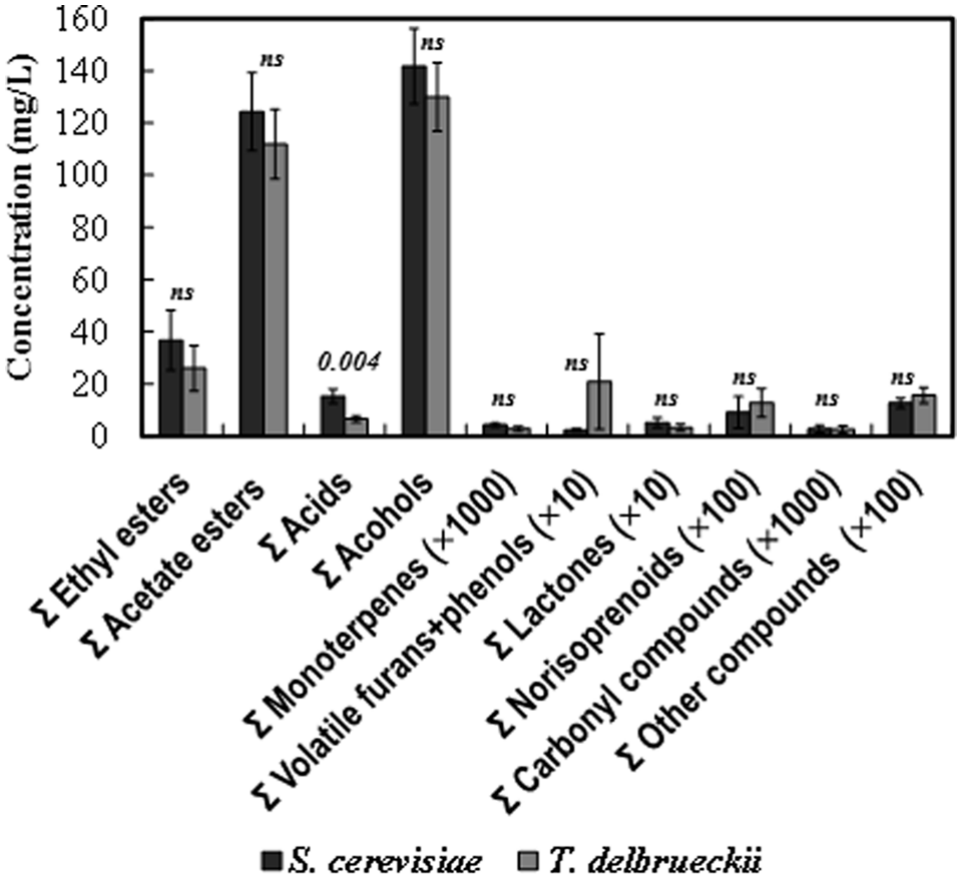
**Aroma compound composition of the *S. cerevisiae*-dominated or *T. delbrueckii*-dominated wines.** The amounts for the similar chemical compounds were pooled as summatory. The data are the mean ± standard error of 23 independent vinifications made in duplicate, 11 inoculated with *S. cerevisiae* and 12 with *T. delbrueckii*. Statistically significant difference *(p)* is stated in the top of the bars. *ns*, no significant difference.

**FIGURE 5:**
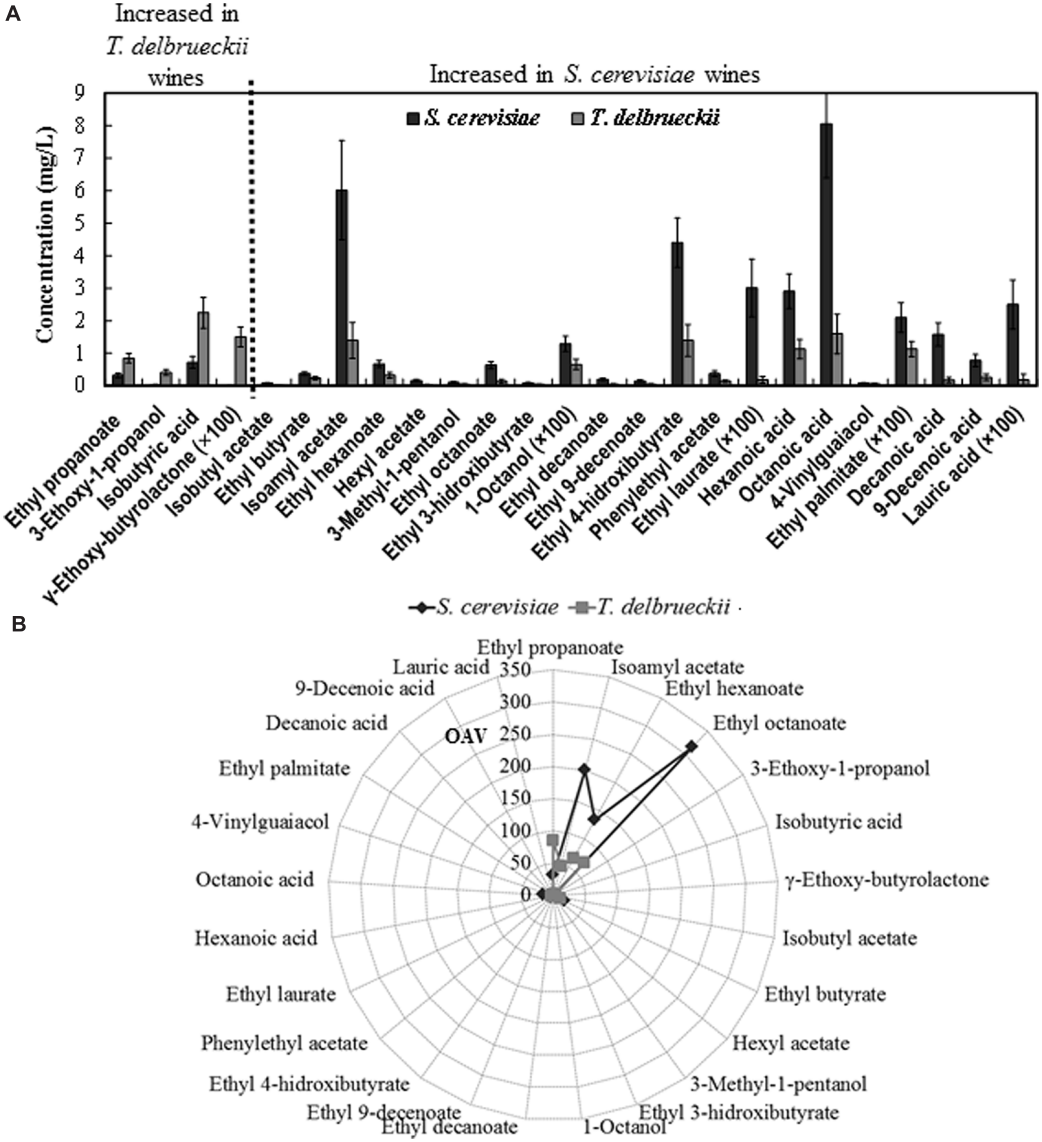
**(A)** Aromatic compounds from which statistically significant difference (*p* < 0.05) were found between the *S. cerevisiae*-dominated and *T. delbrueckii*-dominated wines. The data are the mean ± standard error of 23 independent vinifications made in duplicate, 11 inoculated with *S. cerevisiae* and 12 with *T. delbrueckii*. **(B)** Mean values of the odorant activity values (OAV) for the same compounds in *S. cerevisiae*-dominated and *T. delbrueckii*-dominated white wines.

The total (summatory) amount of ethyl esters, acetate esters, organic acids, alcohols, monoterpenes, lactones, and carbonyl compounds was greater in the *S. cerevisiae* than in the *T. delbrueckii* wines, while the contrary was the case for the amount of furans + volatile phenols and norisoprenoid compounds. However, only the difference found for the amount of organic acids was statistically significant (**Figure 4**). Nevertheless, significant differences were found for 25 of the 75 volatile compounds analyzed independently (**Figure 5**). Only the amounts of ethyl propanoate (odor descriptor: banana, apple), 3-ethoxy-1-propanol (fruity), γ-ethoxy-butyrolactone (as with other lactones, probably cooked peach, coconut, caramel, or toasty odor notes), and isobutyric acid (cheese, sour, butter) were significantly greater in *T. delbrueckii* than in *S. cerevisiae* wines. In contrast, most compounds were more abundant in *S. cerevisiae* than in *T. delbrueckii* wines. These were principally ethyl esters (e.g., ethyl butyrate, ethyl hexanoate, ethyl octanoate, ethyl 3-hydroxybutyrate, ethyl decanoate, ethyl 9-decenoate, ethyl 4-hydroxybutyrate, ethyl laurate, ethyl palmitate) or acetate esters (e.g., isobutyl acetate, isoamyl acetate, hexyl acetate, and phenylethyl acetate), all with fresh fruit odors (**Figure 5A**). Taking the detection thresholds of these 25 aromatic compounds into account, the greatest OAVs corresponded to three compounds with fresh fruit odor descriptors that were more abundant in the *S. cerevisiae* than in the *T. delbrueckii* wines: isoamyl acetate (banana), ethyl hexanoate (banana, green apple), and ethyl octanoate (banana, pineapple, pear, floral; **Figure 5B**). No significant differences were found for the 75 compounds analyzed between the wines from *T. delbrueckii*-non-dominated and *S. cerevisiae*-dominated fermentations (data not shown).

## Discussion

### Influence of *T. delbrueckii* Killer Yeasts on the Must Fermentation Process

The new *T. delbrueckii* killer yeasts were reliably monitored during must fermentation by using spontaneous cyh^R^ mutants, with the results being validated by complementary methods based on molecular polymorphisms or yeast cell morphology. In particular therefore, the results showing that the *T. delbrueckii* Kbarr-1 strain dominated the low-turbidity (<100 NTU) sterile must fermentation when co-inoculated in a 90% initial proportion with 10% of *S. cerevisiae* wine strains are reliable. This initial proportion was much greater than that required for the *S. cerevisiae* killer K2 strain to dominate must fermentation (Pérez et al., 2001), probably because of the faster growth and fermentation rates of *S. cerevisiae* relative to *T. delbrueckii* (Mauricio et al., 1998). Increased must turbidity to values that are frequent in industrial wineries (100–250 NTU) had no relevant inhibitory effect on this *T. delbrueckii* Kbarr-1 domination, and, in particular, much less than the inhibitory effect that had been found previously using *S. cerevisiae* killer-K2 strains (Pérez et al., 2001). This is probably because the *T. delbrueckii* Kbarr-1 strains had a more intense killer phenotype than the *S. cerevisiae* killer-K2 strains (data not shown), and the proportion of the Kbarr-1 toxin that remained unabsorbed onto the grape particles in the turbid must was active enough to kill the 10% of inoculated *S. cerevisiae* yeast. This *T. delbrueckii* Kbarr-1 domination decreased or disappeared when the initial proportion was reduced to 50%, or when the *T. delbrueckii* strain became non-killer. Thus, although the Kbarr-1 killer toxin kills *S. cerevisiae* and helps *T. delbrueckii* Kbarr-1 yeasts to dominate must fermentation, a high initial proportion of *T. delbrueckii* (90%) is required to overcome the greater growth rate of *S. cerevisiae* in the environmental conditions of the present study.

Sequential yeast inoculation with *T. delbrueckii* followed by *S. cerevisiae* did not ensure that the *T. delbrueckii* domination would continue beyond the first 2 days of fermentation. Most often the viable *T. delbrueckii* population quickly fell to less than 10% of total viable yeast cells, the aromatic wine profile was similar to those wines which were single-inoculated with *S. cerevisiae*, and the wines were often not fully dry. As has been shown for assimilable nitrogen limitation (Taillandier et al., 2014), the interference of the growths of the two yeasts could make any given yeast nutrient critically scarce, with the result that the *S. cerevisiae* population is unable to complete must fermentation under this limiting situation. Therefore, this sequential inoculation strategy does not seem appropriate for winemaking because it does not guarantee any relevant and reproducible effect of *T. delbrueckii* on wine quality.

Single *T. delbrueckii* inoculation allowed killer strains to dominate fresh-must fermentation (100–75%), but not the non-killer strains. The *T. delbrueckii*–dominated fermentations were rather slow at the end, and the resulting wines usually contained some reducing sugars. This was not a relevant issue, however, because part of this sugar was metabolized to reach wine dryness after 20–30 days of wine maturation (data not shown). The presence of low amounts of viable *S. cerevisiae* ethanol-resistant wild yeasts seems to ensure completion of the fermentation to give dry wines. This could be because there is none of the aforementioned two-yeast-growth interference at this maturation stage since most of the *T. delbrueckii* cells are dead and cannot secrete the required amount of active killer toxin to kill the ethanol-resistant *S. cerevisiae* cells. None of these wines presented malolactic fermentation as long as the musts were thoroughly clarified and their pH was 3.3 or lower. However, the *T. delbrueckii*–dominated wine from the same musts containing more grape particles and pH above 3.5 underwent malolactic fermentation, which is usually undesirable in white table wines. This was probably because of the larger wild bacteria population associated with the solid particles of the turbid must, and because that a pH above 3.5 did not greatly restrict the growth of lactic acid bacteria.

### Influence of *T. delbrueckii* Killer Yeasts on the Organoleptic Quality and Aroma Compounds of the Wines

The main fermentation aroma of the *T. delbrueckii*–dominated fermentations and the resulting wines, dried/cooked fruit and pastry/candy, did not appear in the wines from *T. delbrueckii*–non-dominated fermentations, which were very similar to those from *S. cerevisiae*–dominated fermentations, fresh fruit aroma, as usual for young white wines. These results were coherent with the significant differences in the content of 25 aroma compounds found in the two wine types. Most of the compounds with fresh fruit odor descriptors were more abundant in the *S. cerevisiae*–dominated wines, including those with the greatest OAVs: isoamyl acetate, ethyl hexanoate, and ethyl octanoate (**Figure 5B**). However, no significant differences were found for the sum of compounds believed to be responsible for a dried/cooked fruit aroma, such as lactones (Hernandez-Orte et al., 2008; Azzolini et al., 2012; **Figure 4**), although a significantly greater amount of γ-ethoxy-butyrolactone was found in the *T. delbrueckii*–dominated than in the *S. cerevisiae*–dominated wines (**Figure 5**). However, a greater amount of ethyl 4-hydroxybutanoate (meringue) was detected in the *S. cerevisiae*–dominated wines. While this can potentially be responsible for some pastry odor, no such odor was detected in these wines by the trained judges. An explanation for these apparently contradictory results could be that, in the *S. cerevisiae*–dominated wines, the main compounds which had fresh-fruit-odor descriptors overcame the possibility of detecting the minor compounds which had dried fruit or pastry odor descriptors. On the contrary, the relative low concentrations of fresh-fruit-odor compounds in the *T. delbrueckii*–dominated wines made it possible to detect the dried fruit and pastry odors. Additionally, the slightly sour and evolved/aged flavor detected in the *T. delbrueckii*–dominated wines but not in the *S. cerevisiae*–dominated wines may have been due to the greater isobutyric acid concentration in the former (**Figure 5**), and which would be coherent with previous findings (Herraiz et al., 1990).

Overall, our results are partially in agreement with those previously reported for the influence of *T. delbrueckii* on the wine quality and aroma compound concentrations. The appearance of dried fruit/coconut aromas associated with the increase in some lactones and the decrease in some ethyl and acetate esters has also been observed in *T. delbrueckii* wine from synthetic white must (Hernandez-Orte et al., 2008). Similarly, the decrease in isoamyl acetate and ethyl esters of C_4_–C_10_ fatty acids has also been noted in *T. delbrueckii* dry white wine from Soave and Chardonnay grape musts, as well as in sweet “Vino Santo” wine from dried Nosiola grapes (Azzolini et al., 2015), although increases in lactones were found only in this last case. Also similarly to our results, that work’s *T. delbrueckii* dry wine had significantly lower freshness and acidity but higher flavor intensity, complexity, and persistence than the *S. cerevisiae* wines. The increased amount of lactones in the “Vino Santo” dessert wine was assumed to improve its organoleptic quality, although this point was not confirmed (Azzolini et al., 2015). Most esters were also found at much lower concentrations in *T. delbrueckii* than in *S. cerevisiae* Sauvignon Blanc dry wines (Renault et al., 2015), although some “minor” esters were considered as produced preferentially by *T. delbrueckii*, in particular ethyl propanoate (in agreement with our findings), ethyl isobutanoate, and ethyl dihydrocinnamate. Additionally, but contrary to our findings, isobutyl acetate and isoamyl acetate concentrations were systematically greater with mixed *T. delbrueckii*/*S. cerevisiae* inoculation although this increase did not correlate with the growth of either species, suggesting that this ester concentration enhancement was due to *S. cerevisiae* production in response to the presence of *T. delbrueckii* (Renault et al., 2015). This increase in isoamyl acetate (banana note) related to *T. delbrueckii* inoculation is rather unexpected given that the contrary has been reported several times (Comitini et al., 2011; Azzolini et al., 2012; Sadoudi et al., 2012), and there has also been a report of greater hydrolytic activity of isoamyl acetate (via esterase) with *T. delbrueckii* than with *S. cerevisiae* (Plata et al., 2003). In contrast, the increase in ethyl propanoate, ethyl isobutanoate, and ethyl dihydrocinnamate is in agreement with previous findings (Herraiz et al., 1990; Moreno et al., 1991; Plata et al., 2003; Hernandez-Orte et al., 2008; Renault et al., 2009; Sadoudi et al., 2012) and with this present work for the case of ethyl propanoate. One can find additional apparent disagreements in the literature for the relative amounts of other compounds produced by *T. delbrueckii* relative to *S. cerevisiae*, but those compounds are not thought to be as relevant for wine aroma as the aforementioned esters and lactones. Although these disagreements could be due to the different yeast strains inoculated in the winemaking (Renault et al., 2009), we did not find any significant differences among the *T. delbrueckii* strains used in this present study (data not shown). Therefore, we think that other vinification parameters are responsible for the disagreements, especially the degree of dominance of the inoculated yeasts because the *S. cerevisiae*-dominated wines had similar aroma profiles independently of whether or not they had previously been inoculated and partially fermented with *T. delbrueckii*. Only the wines from those vinifications inoculated and clearly dominated by *T. delbrueckii* had a differentiated aroma profile. We cannot evaluate the possible influence of the occurrence of malolactic fermentation on the *T. delbrueckii* wine because this aspect has as yet to be taken into account in any depth in previous studies.

In sum, it seems that *T. delbrueckii* has some common effects on wine quality and aroma composition independently of the winemaking condition as long as it is the most relevant yeast species during fermentation. These effects are reduction of the main ester concentrations, increase of some minor ethyl esters and lactone concentrations, and reduction of fresh fruit aromas. However, this yeast can lead to the production of some interesting wine aromas depending on the must type, the yeast inoculation procedure, the degree of the inoculated yeast’s dominance, yeast strain, etc. This variable behavior may determine the wine quality score given by the judges in the sensory evaluation. Therefore, further research on this topic is required to determine the best procedure for the use of *T. delbrueckii* at winery level in order to ensure the expected effect of this yeast on commercial wines’ complexity.

Notwithstanding this finding of variability in the *T. delbrueckii* wine aroma composition, a clear conclusion that can be drawn from this work is that the new *T. delbrueckii* killer strains had the additional advantage of dominating must fermentation in the presence of *S. cerevisiae* relative to the non-killer strains. They significantly decreased the amounts of the main ethyl and acetate ester compounds responsible for a fresh fruit wine aroma, while increasing some minor ethyl ester and lactone compounds that may be responsible for an improved wine complexity. These killer yeasts can be easily and reliably monitored during must fermentation by the incorporated cyh^R^ genetic marker, cell/spore morphology, or molecular polymorphism analyses. Also, they were able to complete the must fermentation of white wines with less than 11.5% ethanol when single inoculated in low-turbidity low-pH must without favoring the growth of lactic acid bacteria.

## Author Contributions

MR conceived the project. MR, RV, MÁ, and EZ designed and performed the experiments. MR, RV, EZ, and LH analyzed the data. MR wrote and edited the manuscript.

## Conflict of InterestStatement

The authors declare that the research was conducted in the absence of any commercial or financial relationships that could be construed as a potential conflict of interest.
