# Supplementary material for: Effects of new Torulaspora delbrueckii killer yeasts on the must fermentation kinetics and aroma compounds of white table wine
Source: Front Microbiol. 2015 Nov 3;6:1222. doi: 10.3389/fmicb.2015.01222 (PMC4630308; doi:10.3389/fmicb.2015.01222)
Supplement: Supplementary file 1 [file Presentation_1.PDF]

Supplementary Material

Effects of new *Torulaspora delbrueckii* killer yeasts on the must fermentation kinetics and aroma compounds of white table wine

Rocío Velázquez, Emiliano Zamora, María L. Álvarez, Luis M. Hernández and Manuel Ramírez*

*** Correspondence:** mramirez@unex.es

1.1 Supplementary Figures

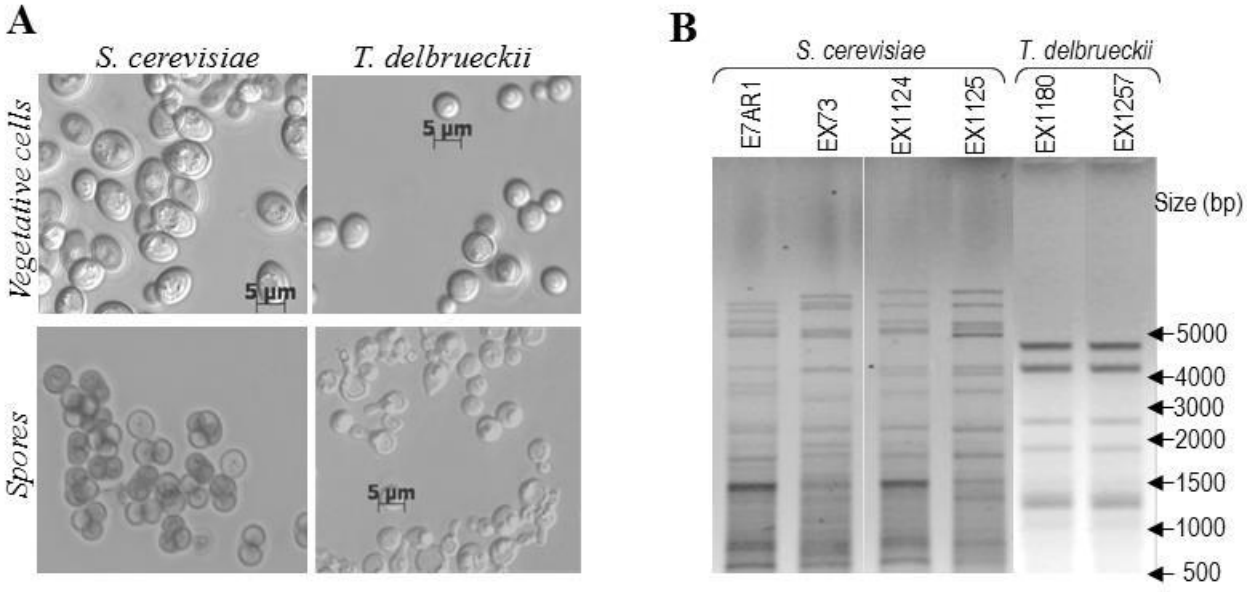

Supplementary Figure 1. A: Microscopic observation (600×) of vegetative cells (top panels) and spores (bottom panels) of *S. cerevisiae* (larger ellipsoidal cell shapes and typical tetrad shaped ascospores) and *T. delbrueckii* (see smaller rounded cell shape and conjugative tubes instead of typical tetrad shaped ascospores). B: Mitochondrial DNA restriction analysis with restriction endonuclease RsaI of several *S. cerevisiae* and *T. delbrueckii* strains (note that the largest DNA bands, greater than 5000 bp, are absent in *T. delbrueckii* strains). Markers: 1 kb DNA Ladder from Biotools.

1.2 Supplementary Tables

Supplementary Table 1. Volatile compounds found in the wines produced (CAS number, retention time, and commercial source for the authenticated standard).

Compound	CAS	RT	Company	Compound	CAS	RT	Company
**Ethyl esters**							
Ethyl propanoate	105-37-3	8.9	SA	1-Pentanol	71-41-0	27	AA
Ethyl isobutyrate	97-62-1	9.1	SA	3-Methyl-1-pentanol	589-35-5	35.2	SA
Ethyl butyrate	105-54-4	11.8	AA	1-Hexanol	111-27-31	38.6	AA
Ethyl 2-methylbutyrate	7452-79-1	12.58	SA	3-Hexen-1-ol	928-96-1	40.5	AA
Ethyl isovalerate	108-64-5	13.4	AA	3-Ethoxy-1-propanol	111-35-3	42.5	SA
Ethyl hexanoate	123-66-0	25.2	SA	Cis-3-hexenol	928-96-1	43.5	AA
Ethyl lactate	97-64-3	38.1	SA	1-Octanol	111-87-5	60.8	AA
Ethyl octanoate	106-32-1	48.5	SA	Methionol	505-10-2	73.1	SA
Ethyl 3-hydroxybutyrate	5405-41-4	58	SA	Benzyl alcohol	100-51-6	83	SA
Ethyl 2-hydroxyhexanoate	52089-55-1	59.9	SA	Phenethyl alcohol	60-12-8	85	AA
Isoamyl lactate	19329-89-6	62.1	TCI	**Monoterpenes**			
Ethyl decanoate	110-38-3	66.4	SA	Linalool	78-70-6	59.8	SA
Ethyl 2-furoate	614-99-3	66.7	SA	α-Terpineol	98-55-5	71.27	AA
Diethyl succinate	123-25-1	69.8	AA	Geraniol	106-24-1	80.3	AA
Ethyl 9-decenoate	67233-91-4	70.7	-	**Volatile furans + phenols**			
Ethyl 4-hydroxybutanoate	999-10-0	78.3	ALC	Furfural	98-01-1	53.5	SA
Ethyl laurate	106-33-2	80	SA	4-Hydroxy-2,5-dimethyl-3(2H)furanone	3658-77-3	90.98	M
Diethyl malate	7554-12-3	91.5	SA	4-Vinylguaiacol	7786-61-0	101	SA
Diethyl 2-hydroxyglutarate	69134-53-8	98	-	2,4-di-tert-butyl phenol	96-76-4	108.2	SA
Ethyl palmitate	628-97-7	103	SA	4-Vinylphenol	2628-17-3	117.9	SA
Ethyl 2-hydroxy-3-phenylpropanoate	15399-05-0	106.7	-	**Lactones**			
Ethyl succinate	1070-34-4	116.6	AA	γ-Butyrolactone	96-48-0	68	SA
**Acetate esters**				γ-Hexalactone	695-06-7	72.44	SA
Ethyl acetate	141-78-6	7.6	SA	γ-Ethoxy-butyrolactone	932-85-4	74.3	-
Isobutyl acetate	110-19-0	10.79	AA	Whiskey lactone	39212-23-2	83.53	SA
Isoamyl acetate	123-92-2	16.6	AA	γ-Nonanolactone	104-61-0	91.2	SA
Hexyl acetate	142-92-7	29.3	SA	Solerone	29393-32-6	93.25	SA
Phenethyl acetate	103-45-7	79.1	SA	γ-Decalactone	706-14-9	98.24	AA
**Acids**				Sotolon	28664-35-9	100.58	SA
Isobutyric acid	79-31-2	63.1	SCA	δ-Decalactone	705-86-2	100.6	SA
Butanoic acid	107-92-6	67.6	SA	Ethoxycarbonyl-γ-butyrolactone	33019-03-3	103.9	SA
Isovaleric acid	503-74-2	70.4	AA	γ-Dodecalactone	706-14-9	114.94	SA
Hexanoic acid	142-62-1	81.1	AA	**Norisoprenoids**			
Trans-2-Hexenoic acid	13419-69-7	87.9	AA	β-Damascenone	23726-93-4	79.32	SA
Octanoic acid	124-07-2	92.3	SA	**Carbonyl compounds**			
Decanoic acid	334-48-5	105.2	SA	Acetoin	513-86-0	31.8	SA
9-Decenoic acid	14436-32-9	110.6	SA	**Other compounds**			
Lauric acid	143-07-7	127	AA	1,3-di-tert-butylbenzene	1014-60-4	47.6	SA
**Alcohols**				N-pentadecane	629-62-9	53.5	AA
Isobutanol	78-83-1	14.5	SA	Blackberry thiophenone	13679-85-1	59.4	SA
1-Butanol	71-36-31	18.2	SA	Phenyl acetaldehyde	122-78-1	68.6	AA
Isoamyl alcohol	123-51-3	23.1	SA	N-(3-methylbutyl) acetamide	13434-12-3	81.7	FC

SA, Sigma-Aldrich. AA, Alfa Aesar. TCI, Tokyo Chemical Industry. ALC, AldLab Chemical. SCA, SuperCo Analytical. M, Merck. FC, FluoroChem Ltd.
